# The Fascination of Opthalmology

**Published:** 1913-02

**Authors:** Cecil Stockard

**Affiliations:** Atlanta, Ga.


### THE FASCINATION OF OPHTHALMOLOGY

By Cecil Stockard, M. D., Atlanta, Ga.

This paper is not, strictly speaking, scientific; in fact, it may be said to deal rather with the sentimental side of special practice.

I believe that the best surgeon is not, necessarily, the one who knows most about surgery, but rather the one who loves surgery best, and who is most fascinated by that line of work. Although, usually, as a matter of fact, the one who likes a subject is naturally the one who knows most about it. This is true of course, of any branch of medicine, and I think it is of some value to know why the surgeon likes surgery, the neurologist, fact, we may go still further and say that no man can be truly successful who does not love his lifework with all his heart.

There is good reason for the excellent advice given a young neurology; the dermatologist, dermatology; and so on, and it is my purpose in this paper to tell some of the reasons why an ophthalmologist likes ophthalomology.

An ancient poem on happiness gives a list of the joys of the simple life followed by the statement that

"—— all this,
And the love of a peaceful calling,
Truly is greatest bliss."

It is certainly true that no man can get his full share of happiness out of life who does not thoroughly love his lifework; in friend of mine who was planning to study for the ministry. He naturally went to old and experienced preachers for advice, and one of them said to him, "Robert, if you can't love the ministry just like you would a girl, you would better try something else."

This rule applies to any work, but is especially true of our profession. No one has a right to be responsible for the life and health of his fellows, who does not really love his work.

In speaking of the fascinating features of ophthalmic practice I do not pretend to assume that it is the most interesting and important phase of the practice of medicine; however true that may be to my mind, I do not expect everyone to agree with me; but I merely wish to discuss some of the things that lend zest to the work of an ophthalmologist.

It is always a pleasure to make a diagnosis, a satisfaction to get results, and when, as sometimes happens, the patient is really grateful, the doctor feels that his work is worthwhile after all. This applies to any branch of medicine, as do some other things I shall say; but there are some things that are peculiar to ophthalmology.

There are many interesting features about refractive work; the marvellous improvement in vision, and the great relief of eye strain and headaches that accompany the adjustment of proper lenses, seem at times like magic, and indeed they are magic in the truest and best sense of the word.

It has not been many years since astigmatism was an unknown subject, and patients suffering from this condition were literally "a stigma" or without a focal point even with the best lenses that could be fitted for them; but the use of cylindrical lenses has made it possible to give many of these cases the same relief that can be more easily given hyperopes and myopes.

Retinoscopy is one of the most interesting and at the same time most accurate means of diagnosis in all the realm of medicine. By this method children and idiots may be refracted with no subjective assistance whatever.

The prescriber of lenses has a great advantage over the prescriber of drugs in that the prescription can be tested and the slightest discrepancy detected in every case, whereas, when drugs are prescribed one usually has only the druggist's word and reputation to fall back on, consequently substitution is much easier.

The many advances and improvements in lenses that have been made in recent years have led dreamers to imagine still further improvements, and perhaps the most striking of these dreams is that of contact lenses—lenses that shall be worn in actual contact with the cornea, or practically so. The optical advantages of this scheme are apparent, and who shall say that it is impossible? Still wilder dreams than this have been realized in the past, so why should this remain always only a dream?

There are a large number of commoner eye conditions in which anyone with a proper training can be surer of his diagnosis than in perhaps any other branch of medicine. A diagnosis can usually be made more quickly than anywhere else, except possibly the skin; and in making a diagnosis, (even in refractive errors as far as the use of the ophthalmoscope and retimoscope are concerned,) we have to rely almost entirely on visible symptoms, usually getting but little assistance from subjective symptoms and history. In a large majority of conditions no further examination is necessary, no percussion, no anscultation, rarely any laboratory work, and palpation only to detect glaucoma or tumors. Thus, as the diagnosis depends largely on the use of the eyes, it is possible to reach such a stage of perfection as to be able to examine an eye at a glance and then describe everything that is to be seen. Training here is, of course, of paramount inportance.

I remember once when I was a student I was given a clinical patient to examine.. I had been giving considerable attention to this line of work, and was fairly familiar with the commoner diseases, but after making what I thought was a thorough examination, I was still unable to find a cause for the pain complained of by the patient, and had to be shown the end of an eyelash which was caught in the superior lacrymal punctum, where a better trained eye saw it almost immediately.

The important relationship that the eye bears to general disease adds interest to its consideration. The famous remark of Helmhotz, who said, when he first looked through the ophthalmoscope into the human eye, that he could see the man's soul, is not altogether fanciful. The things that one can see are at times astounding. Recently I made a diagnosis of Bright's disease in a case in which the vision was about normal, and the only complaint was eyestrain and headaches. The ophthalmoscope showed a small snowbank patch, about 4mm. long and 2 mm. wide just above the optic disc in each eye, the rest of the eye-ground being normal. The diagnosis was confirmed by Dr. G. L. Bush who now has the patient under observation. In a case like this the oculist is able to make a direct visual examination of the small blood vessels of the type chiefly involved.

Almost any association of ocular and general disease presents interesting features for study. For example, the presence or absence of choke disc is of great diagnostic value in suspected tumor of the base of the brain, and the presence of albuminurea of pregnancy is an indication for abortion, and its detection is of greatest importance.

Ophthalmic surgery is in a class by itself, both as to the delicacy of the operations, and the brilliancy of immediate results. An abdominal operation may save a patient's life, but at best he is no better off than before; but it is difficult to imagine a greater change in one's whole attitude toward life than frequently follows the restoration of sight to one who has been blind for several years from cataracts, for instance. And even the removal of a foreign body from the cornea is often accompanied by a relief of pain that is almost unparalleled. Some time ago a girl was brought to me, sixteen years old, cross-eyed, and naturally very timid and retiring. A simple anotomy corrected her squint, and in a few months she was the belle of the town. What other branch of surgery can produce results like that?

Most of the drugs used in ophthalmology have well known definite therapeutic action, so, when one knows just what effect is desired, it is comparatively easy to choose a remedial agent that will usually produce that effect, and it is very rarely necessary to give a "shotgun" prescription. This reduces guesswork therapentics to a minimum, and is one of the most fascinating features of the whole subject.

This possibility of therapeutic precision also makes it possible, frequently, in a case that has been treated by another doctor, to decide from the treatment whether your predecessor has made a correct diagnosis. I had a case recently that well illustrates this. I was called to see an old lady seventy-odd years old, who had been suffering with her eyes for two weeks, and I found that the doctor had given her a cocain solution to use locally, and aspirin internally; that was all. The poor old soul was suffering torture from absolute glaucoma in both eyes, and her family physician had never suspected it.

This case emphasizes the importance of specialties in medicine. No one man should try to keep up with all branches of medicine, for, to paraphrase Barmun, "You can know all about something, or something about everything, but you can't know all about everything."

One will instinctively pay more and more attention to the thing he does most of, and likes and will have a tendency to neglect the things that do not fascinate him. We hear a great deal about preachers being "called," and I think a specialist should be called too. This call consists, of course, in the fascination, and irresistible leaning toward a particular line of work—and if a man will follow this leaning, and obey this call he will have the greatest possible chance to do the greatest possible good, and to attain to the greatest possible success.

903-5 Empire Life Building.

